# Reviewing harm reduction for people who inject drugs in Asia: the necessity for growth

**DOI:** 10.1186/s12954-015-0066-x

**Published:** 2015-10-16

**Authors:** Katie Alexandra Stone

**Affiliations:** Harm Reduction International, Unit 2C09, South Bank Technopark, 90 London Road, London, SE1 6LN UK

**Keywords:** Harm reduction, Review, Asia, People who inject drugs (PWID), Needle and syringe programmes (NSP), Opioid substitution treatment (OST), Antiretroviral treatment (ART), Tuberculosis (TB)

## Abstract

**Background:**

There is an estimate of three to five million people who inject drugs living in Asia. Unsafe injecting drug use is a major driver of both the HIV and hepatitis C (HCV) epidemic in this region, and an increase in incidence among people who inject drugs continues. Although harm reduction is becoming increasingly accepted, a largely punitive policy remains firmly in place, undermining access to life-saving programmes. The aim of this study is to present an overview of key findings on harm reduction in Asia based on data collected for the Global State of Harm Reduction 2014.

**Methods:**

A review of international scientific and grey literature was undertaken between May and September 2014, including reports from multilateral agencies and international non-governmental organisations. A qualitative survey comprising open-ended questions was also administered to civil society, harm reduction networks, and organisations of people who use drugs to obtain national and regional information on key developments in harm reduction. Expert consultation from academics and key thinkers on HIV, drug use, and harm reduction was used to verify findings.

**Results:**

In 2014, 17 countries in Asia provide needle and syringe programmes (NSP) provision and 15 opioid substitution therapy (OST). It is estimated that between 60 and 90 % of people who use drugs in Asia have HCV; however, treatment still remains out of reach due to cost barriers. TB testing and treatment services are yet to be established for key populations, yet nearly 15 % of the global burden of new cases of HIV-TB co-infection are attributed to southeast Asia. Eighteen percent of the total number of people living with HIV eligible for antiretroviral treatment (ART) accessed treatment. Only Malaysia and Indonesia provide OST in prison, with no NSP provision in prisons in the region.

**Conclusion:**

To reduce HIV and viral hepatitis risk among people who inject drugs, there is a necessity to significantly increase harm reduction service provision in Asia. Although there has been progress, work still needs to be done to ensure an appropriate and enabling environment. At present, people who inject drugs are extremely difficult to reach; structural and legal barriers to services must be reduced, integrated holistic services introduced, and further research undertaken.

**Electronic supplementary material:**

The online version of this article (doi:10.1186/s12954-015-0066-x) contains supplementary material, which is available to authorized users.

## Background

Injecting drug use has been documented in at least 158 countries and territories worldwide [1]. Although harm reduction is becoming increasingly accepted, further initiatives are vital for its successful implementation in Asia. Asia is one of the regions particularly affected by HIV among people who inject drugs [2], with an estimated three to five million people who inject drugs living in the region [3]. HIV prevalence among people who use drugs ranges from over 40 % in the Philippines [4] to under 10 % in China [5]. Asia, however, remains a region which relies upon an overly-punitive response to drugs, for example it is still one of the few areas of the globe that continue to implement the death penalty for drug offenses, and 11 countries continue to use compulsory centres for people who use drugs as their primary approach to treatment [3]. These types of services have been shown to be both ineffective [6, 7] and unethical [8], yet countries such as Cambodia, Thailand, and Vietnam have yet to amend laws relating to detention centres.

In places where harm reduction has been brought to sufficient scale and quality, it has had a significant impact in reducing HIV transmission. For example, Nepal, which was an early implementer of harm reduction in Asia, saw rates of HIV prevalence decline drastically from 68 % in 2002 to 6.3 % in 2011 [3]. In a study undertaken in Southwest China, the HIV incident rate among people who inject drugs dropped significantly from 2.5 to 0.6 cases per 100 person-years after the implementation of harm reduction strategies including NSP, OST, counselling, and condom promotion [9]. And a further study in Taiwan, among people who inject drugs released from prison, also found a decrease in HIV incidence from 18.2 % in 2005 to 0.3 % in 2010, associated with attendance at methadone clinics and frequency of NSP attendance [10].

Although the evidence for harm reduction, particularly the use of NSP, OST, HIV testing and counselling and anti-retroviral therapy as recommended by the World Health Organization (WHO), United Nations Office on Drugs and Crime (UNODC) and the Joint United Nations Programme on HIV and AIDS (UNAIDS) [11], has been widely acknowledged as a best practice approach, there are still countries in Asia where a primarily punitive policy and legal environment blocks access for people who use drugs to these services, and provision is at best lacking and at worst non-existent.

This paper looks at harm reduction responses in Asia focusing on the impact a lack of services can have on HIV, viral hepatitis, and TB epidemics. Using figures from the Global State of Harm Reduction report 2014 [12] (see Additional file 1: Table S1), this paper will illustrate the ways in which policy has been successfully translated into practice, countries which have struggled to implement harm reduction irrespective of policy decisions, and places in which punitive regimes and moral concerns have overruled empirical evidence increasing both risk and transmission of viruses among people who inject drugs.

## Methods

The basis of the findings from this paper is derived from the Global State of Harm Reduction 2014 report [12]. Harm Reduction International has been mapping the response to drug-related HIV and hepatitis C epidemics around the world since 2008. The information presented in the 2014 report has been gathered using existing data sources, including research papers and reports from multilateral agencies, international non-governmental organisations, civil society and harm reduction networks, organisations of people who use drugs, and expert and academic opinion from those working on HIV, drug use, and harm reduction. The biennial report integrates updated information on harm reduction services, including needle and syringe programmes (NSPs) and opioid substitution therapy (OST) provision, harm reduction services in the prison setting, access to antiretroviral therapy for people who inject drugs; regional overdose responses, policy developments, civil society developments, and information relating to funding for harm reduction. A review of international scientific and grey literature was undertaken between May and September 2014. A qualitative survey, comprising open-ended questions, was administered alongside this to civil society, harm reduction networks, and organisations of people who use drugs to obtain national and regional information on key developments in harm reduction. A narrative analysis was undertaken to synthesise findings, and expert consultation from academics and key thinkers on HIV, drug use, and harm reduction was used to peer-review and verify findings.

## Results

### HIV epidemiology

Additional file 1: Table S1 provides epidemiology from 2014 of HIV, viral hepatitis, and harm reduction responses in Asia. The average rate of HIV prevalence among people who inject drugs in Asia is 15.46 % [12]. HIV among this key population has increased in six countries (Cambodia, Indonesia, Malaysia, Philippines, Taiwan, and Thailand) since 2012 by an average of 7.75 % [12, 13]. However, the greatest growth can be seen in the Philippines, where a 28 % increase in HIV among people who inject drugs was seen between 2012 and 2014 [12, 13]. The HIV epidemic in the Philippines has been steadily growing, and since 2008, has risen by 40 % among people who inject drugs [1, 12, 14, 15]. Given the estimated number of people who inject drugs in the Philippines (mean average 14,456), there is a distinct lack of harm reduction service provision, with only three NSP sites in operation [13] and no OST provision. The lowest HIV prevalence rates in Asia can be seen in Bangladesh, with just 1.1 % of people who inject drugs [16] and 88 NSP and three OST sites in operation as of 2014. This highlights the relative coverage and impact the early implementation of harm reduction services can have.

### Hepatitis C and B epidemiology

In Japan, hepatitis C prevalence among people who inject drugs is 64.8 % [17], and 54 % [17] in the Republic of Korea, yet still no services exist for this marginalised group. Thailand, Pakistan, Nepal, and Macau continue to have over an 80 % hepatitis C prevalence among people who inject drugs [17, 18]. It is important to note that this information is often not regularly collected, meaning data, aside from Macau, dates from prior to 2003. In Asia alone, approximately 2.6 million people (range 1.8–3.6 million) are living with hepatitis C [17], with an average prevalence rate among people who inject drugs at 60.7 % [12]. Significant efforts have been made by civil society organisations and people who use drugs in Indonesia to scale up the government response to hepatitis C treatment, with a set of guidelines becoming available in 2014 laying out plans to integrate hepatitis C treatment into national surveillance among people who inject drugs as of 2015 [19]. However, on the whole, hepatitis treatment still remains out of reach for most people due to exorbitant prices, and urgent measures are needed to ensure that access to diagnosis and treatment improves.

### ART uptake among people who inject drugs

Although there is antiretroviral provision (antiretroviral treatment (ART)) in Asia, more than two-thirds of people who inject drugs do not know their HIV status [3], and only 18 % of people living with HIV eligible for treatment access it in this region [3]. This may be attributed to barriers such as stigmatisation by healthcare staff, a lack of confidentiality, and no HIV testing offered within harm reduction settings [20]. Studies providing evidence of the benefits of integrating OST, HIV testing and counselling, and ART in places such as Vietnam are, however, helping to support and promote the integration of these services through demonstrating higher retention rates than drug users who do not receive integrated service provision [21]. Joint service provision within a harm reduction setting is therefore imperative to ensure people who inject drugs access ART.

### TB among people who inject drugs

Asia accounts for nearly 15 % of the global burden of new cases of HIV-TB co-infection, [22] yet data on TB for people who inject drugs is sparse. Individual studies, however, indicate a disproportionate prevalence of TB among people who inject drugs, with Malaysia reporting TB-HIV co-infection for detainees in closed settings, such as prisons and drug rehabilitation centres, increasing from six to 1477 cases between 1990 and 2013 [23, 24]. Although TB treatment is integrated into HIV programmes in Bhutan, Cambodia, India, Indonesia, and Nepal, it is unknown whether such services are designed for people who inject drugs, and there is still a lack of integration of TB testing and treatment into harm reduction programmes [25].

## Discussion

### Harm reduction responses

Although 19 countries in Asia explicitly include harm reduction in their national plans and drug policies, the existence does not necessarily equate to provision of an adequate response in either scope or quality. Harm reduction interventions in the form of NSP and OST have yet to be established in six countries within Asia. And although injecting drug use is common in prisons in the region [26, 27], only Malaysia and Indonesia provide OST in this setting, with no NSP available. Cambodia’s Drug Control Law 2012 states that a person who injects drugs and is arrested for a drug-related offence should be referred to treatment and related services [28], yet this legal provision of referral is often not implemented [29]. Other countries have seen improvements through passing legal reforms, such as Singapore, who in 2013 passed a law allowing those facing the death penalty the opportunity to ask for resentencing, with nine prisoners applying having their sentences reduced to life imprisonment rather than death [30].

Malaysia is perhaps one of the best examples in Asia in terms of the adoption and acceptance of harm reduction, with signs of increasing political commitment to a gradual shift away from a punitive approach to a voluntary, rights-based approach to drug treatment [25]. Malaysia has transformed eight compulsory detention centres into voluntary cure and care centres or clinics, widely praised as best practice for the region [3]. Malaysia has also increased its OST provision in prisons, expanding from one site in 2008 to 18 by 2013 [23], and its NSP site provision by 431 between 2012 and 2014 [12, 13]. A reduction in HIV prevalence among people who inject drugs is yet to be seen in this country; however, with continued commitment and increasing implementation of services, 18.9 % [23] of people who inject drugs currently living with HIV should steadily decrease.

Thanks to various civil society projects which have been initiated in Asia, a successful response to HIV, viral hepatitis, and TB among people who use drugs is gradually becoming more feasible. Asia Action, a European Commission funded project, has supported AIDS Care China to scale up naloxone training and dissemination among people who inject drugs [25]; in Cambodia, work with police officers to offer a more enabling environment and reduce human rights violations against drug users is underway [28], and research documenting the implementation of Indonesia’s 2011 diversion policy, which stipulates that people arrested on drug-related offences with personal possession under a stated weight should be referred to treatment rather than imprisoned, is in process [31]. The Asian Network of People who Use drugs (ANPUD), representing the voices of communities of people who use drugs in the region, have also played an important role in advocating for evidence-based changes in punitive drug laws and policies [25].

This work has given an overview of the harm reduction approaches in Asia synthesising available evidence. There are, however, limitations to the data. In Asia, HIV prevalence rates can range from over 40 % in countries such as Pakistan [32] to less than 2 % in places such as Macau [33]. However, national figures can often hide significant regional and sub-national variations. For example, national figures for HIV prevalence among people who inject drugs appear to be declining in Indonesia from 42.5 % in 2010 [34, 35] to 36.4 % in 2012 [36], yet in urban centres like Jakarta and Surabaya, HIV prevalence estimates were 56.4 and 48.8 %, respectively [37]. Other limitations to the data include only works produced in English and the omission of quality criteria to grade both published and grey literature.

## Conclusion

Harm reduction interventions in Asia are often hindered by a largely punitive policy, poor service provision, a lack of human resources and funding mechanisms, and poor service integration [38], all of which serve to undermine access to life-saving harm reduction programmes. The United Nations 2011 political declaration on HIV/AIDS targets to reduce the transmission of HIV among people who inject drugs by 50 % by 2015, thus becomes increasingly unrealistic, and unsafe injecting practices continue.

With high rates of hepatitis C among people who inject drugs in Asia, this paper highly recommends greater integration of hepatitis C prevention, education, testing, and treatment services for drug users, alongside ART and OST provision integrated not only in community settings, but also in custodial settings. An increase in harm reduction services is also vital to reduce the steady HIV incidence among this marginalised and stigmatised group.

As several international donors are gradually withdrawing support [25], it is a worrying fact that harm reduction services currently running may be adversely affected. To ensure this does not happen, rigorous assessments detailing the adequacy of national funding to support these initiatives are imperative, further work on scaling up harm reduction programmes necessary, and structural barriers reduced to ensure no drug user gets left behind.

## Additional file

Additional file 1:
**Epidemiology of HIV, viral hepatitis and harm reduction responses in Asia.** DOCX 60.0 KB

## References

[1] Cook C, Kanaef N. The Global State of Harm Reduction 2008: mapping the response to drug-related HIV and hepatitis C epidemics. London: Harm Reduction International; 2008.

[2] Wu Z, Shi CX, Detels R (2013). Addressing injecting drug use in Asia and Eastern Europe. Curr HIV/AIDS Rep.

[3] UNAIDS. HIV in Asia and the Pacific. Geneva: UNAIDS; 2013.

[4] Department of Health. Integrated HIV Behavioral & Serologic Surveillance. Philippines: Department of Health; 2013.

[5] UNAIDS. Global AIDS response progress reporting: China. Geneva: UNAIDS; 2014.

[6] Wolfe D, Saucier R (2010). In rehabilitation’s name? Ending institutionalised cruelty and degrading treatment of people who use drugs. Int J Drug Policy.

[7] Leukefeld C, Tims F, Leukefeld C, Tims F (1988). Compulsory treatment: a review of findings. Compulsory treatment of drug abuse: research and clinical practice.

[8] Hall W, Babor T, Edwards G, Laranjeira R, Marsden J, Miller P (2012). Compulsory detention, forced detoxification and enforced labour are not ethically acceptable or effective ways to treat addiction. Addiction (Abingdon, England).

[9] Ruan Y, Liang S, Zhu J, Li X, Pan SW, Liu Q (2013). Evaluation of harm reduction programs on seroincidence of HIV, hepatitis B and C, and syphilis among intravenous drug users in southwest China. Sex Transm Dis.

[10] Huang Y, Yang J, Nelson K (2014). Changes in HIV incidence among people who inject drugs in Taiwan following introduction of a harm reduction program: a study of two cohorts. PLoS Med.

[11] WHO, UNODC, UNAIDS. Technical guide for countries to set targets for universal access to HIV prevention, treatment and care for injecting drug users. Geneva: WHO, UNODC, UNAIDS; 2009.

[12] Stone K. The Global State of Harm Reduction 2014. London: Harm Reduction International; 2014.

[13] Stoicescu C. The Global State of Harm Reduction 2012: towards an integrated response. London: Harm Reduction International; 2012.

[14] Aceijas C, Stimson GV, Hickman M, Rhodes T (2004). Global overview of injecting drug use and HIV infection among injecting drug users. Aids.

[15] Samonte G, Belimac, J., Feliciano, J. Philippine estimate of the most-at-risk population and people living with HIV. Philippine: Department of Health; 2011.

[16] UNAIDS. Global AIDS response progress reporting: Bangladesh. Geneva: UNAIDS; 2014.

[17] Nelson PK, Mathers BM, Cowie B, Hagan H, Des Jarlais D, Horyniak D (2011). Global epidemiology of hepatitis B and hepatitis C in people who inject drugs: results of systematic reviews. Lancet.

[18] Noguiera A (2012). Personal communicaton with Augusto Noguira, President of the Association for Rehabilitation of Drug Abusers of Macau (ARTM).

[19] Stoicescu C (2013). Indonesia's hidden hepatitis C time bomb.

[20] Augustian E, Sugiharto S. GSHR 2014 survey response. London: Indonesian Drug User Network; 2014.

[21] UNAIDS. Global AIDS response progress reporting: Vietnam. Geneva: UNAIDS; 2014.

[22] WHO, SEARO. TB/HIV in the South-East Asia Region. New Delhi: WHO; 2010.

[23] UNAIDS. Global AIDS response progress reporting: Malaysia. Geneva: UNAIDS; 2014.

[24] Ministry of Health. TB surveillance data. Malaysia: Ministry of Health; 2013.

[25] Stoicescu C. GSHR survey response 2014. London: Harm Reduction International; 2014.

[26] Nasir A, Todd CS, Stanekzai MR, Bautista CT, Botros BA, Scott PT (2011). Prevalence of HIV, hepatitis B and hepatitis C and associated risk behaviours amongst injecting drug users in three Afghan cities. Int J Drug Policy.

[27] National Narcotics Bureau of Statistics. 2004.

[28] Reaksmey H. GSHR 2014 survey response. KHANA; 2014.

[29] KHANA N. Cambodia Integrated Biological Behavioral Surveillance Survey (IBBS). Cambodia: Department of Health; 2012.

[30] International A (2013). Singapore: landmark ruling lifts death penalty for drug offender.

[31] Cemara R (2014). Rumah Cemara Indonesia.

[32] UNAIDS. Global AIDS response progress reporting: Pakistan. Geneva: UNAIDS; 2014.

[33] Agrawal DA (2012). Personal communication with Dr Alok Agrawal, Programme Officer.

[34] Cook C. The global state of harm reduction: key issues for broadening the response. London: Harm Reduction International; 2010.

[35] Mathers BM, Degenhardt L, Phillips B, Wiessing L, Hickman M, Strathdee SA (2008). Global epidemiology of injecting drug use and HIV among people who inject drugs: a systematic review. Lancet.

[36] UNAIDS. Global AIDS response progress reporting: Indonesia. Geneva: UNAIDS; 2012.

[37] UNAIDS. The gap report. Geneva: UNAIDS; 2013.

[38] Boltaev AA, El-Bassel N, Deryabina AP, Terlikbaeva A, Gilbert L, Hunt T (2013). Scaling up HIV prevention efforts targeting people who inject drugs in Central Asia: a review of key challenges and ways forward. Drug Alcohol Depend.

