# Supplementary material for: Reviewing harm reduction for people who inject drugs in Asia: the necessity for growth
Source: Harm Reduct J. 2015 Oct 16;12:32. doi: 10.1186/s12954-015-0066-x (PMC4608313; doi:10.1186/s12954-015-0066-x)
Supplement: Additional file 1: — Epidemiology of HIV, viral hepatitis and harm reduction responses in Asia. DOCX 60.0 KB [file 12954_2015_66_MOESM1_ESM.docx]

| Country/territory with reported injecting drug use | People who inject drugs | HIV prevalence among people who inject drugs (%) | Hepatitis C (anti-HCV) prevalence among people who inject drugs (%) | Hepatitis B (anti-HBsAg) prevalence among people who inject drugs (%) | Harm reduction response | |
| --- | --- | --- | --- | --- | --- | --- |
|  |  |  |  |  | NSP^[[1]](#footnote-1)^ | OST^[[2]](#footnote-2)^ |
| Afghanistan | 20,000 (18,000–23,000)^(^[^1^](#_ENREF_1)^)^ | 4.4^(^[^2^](#_ENREF_2)^)^^[[3]](#footnote-3)^ | 31.2^(^[^2^](#_ENREF_2)^)^ | 6.6^(^[^2^](#_ENREF_2)^)^ | 31^(^[^3^](#_ENREF_3)^)^ | 1^(^[^3^](#_ENREF_3)^)^ |
| Bangladesh | 21,800–23,800^(^[^4^](#_ENREF_4)^)^^[[4]](#footnote-4)^ | 1.1^(^[^4^](#_ENREF_4)^)^ | 39.6^(^[^5^](#_ENREF_5)^)^^[[5]](#footnote-5)^ | 9.4^(^[^6^](#_ENREF_6)^)^ | 88^(^[^4^](#_ENREF_4)^)^ | 3^(^[^4^](#_ENREF_4)^)^ (M) |
| Bhutan | nk | nk | nk | nk | x | x |
| Brunei Darussalam | nk | nk | nk | nk | x | x |
| Cambodia | 1,300 (1,200–2,800)^(^[^7^](#_ENREF_7)^)^ | 24.8^(^[^7^](#_ENREF_7)^)^ | nk | nk | ✓ (2) | ✓ (1) |
| China | 2,580,000^(^[^8^](#_ENREF_8)^)^^[[6]](#footnote-6)^ | 6.3([9](#_ENREF_9)) | 67^(^[^6^](#_ENREF_6)^)^ | 10^(^[^6^](#_ENREF_6)^)^ | 898 | 763 (B, M) |
| Hong Kong | nk | nk | nk | nk | x | ✓ |
| India | 177,000–180,000^(^[^10^](#_ENREF_10)^)^^[[7]](#footnote-7)^ | 7.14^(^[^11^](#_ENREF_11)^)^ | 41^(^[^6^](#_ENREF_6)^)^^[[8]](#footnote-8)^ | 10^(^[^6^](#_ENREF_6)^)^ | 295 | 145^(^[^12^](#_ENREF_12)^)^ (B, M, O) |
| Indonesia | 74,326 (61,901–88,320)^(^[^13^](#_ENREF_13)^)^ | 36.4^(^[^14^](#_ENREF_14)^)^ | 77.3^(^[^6^](#_ENREF_6)^)^ | 2.9^(^[^6^](#_ENREF_6)^)^ | 194 | 85 (B, M) |
| Japan | nk | nk | 64.8 (55–74.5)^(^[^6^](#_ENREF_6)^)^ | 3.2 (2–4.3)^(^[^6^](#_ENREF_6)^)^ | x | x |
| Korea (Republic of) | nk | nk | 54^(^[^6^](#_ENREF_6)^)^ | 4^(^[^6^](#_ENREF_6)^)^ | x | x |
| Laos PDR | 1,700^(^[^15^](#_ENREF_15)^)^ | nk | nk | nk | 4^(^[^15^](#_ENREF_15)^)^^[[9]](#footnote-9)^ | x |
| Macau | 238^(^[^16^](#_ENREF_16)^)^ | 1.32^(^[^17^](#_ENREF_17)^)^ | 80.4^(^[^18^](#_ENREF_18)^)^ | 10.7^(^[^18^](#_ENREF_18)^)^ | 4^(^[^18^](#_ENREF_18)^)^ | 4^(^[^18^](#_ENREF_18)^)^ (B, M) |
| Malaysia | 170,000^(^[^19^](#_ENREF_19)^)^ | 18.9^(^[^20^](#_ENREF_20)^)^ | 67.1^(^[^6^](#_ENREF_6)^)^ | ? | 728^(^[^20^](#_ENREF_20)^)^ | 811^(^[^20^](#_ENREF_20)^)^ (B, M) |
| Maldives | 793 (690–896)^(^[^21^](#_ENREF_21)^)^ | 0^(^[^21^](#_ENREF_21)^)^ | 0.7^(^[^22^](#_ENREF_22)^)^ | 0.8^(^[^22^](#_ENREF_22)^)^ | x | 1^(^[^23^](#_ENREF_23)^)^ (M) |
| Mongolia | nk | nk | nk | nk | 1^(^[^24^](#_ENREF_24)^)^ | x |
| Myanmar | 75,000^(^[^25^](#_ENREF_25)^)^ | 18.7^(^[^26^](#_ENREF_26)^)^ | 79.2^(^[^6^](#_ENREF_6)^)^ | 9.1^(^[^6^](#_ENREF_6)^)^ | 40^(^[^25^](#_ENREF_25)^)^ | 18^(^[^25^](#_ENREF_25)^)^ |
| Nepal | 52,174^(^[^27^](#_ENREF_27)^)^ | 6.3^(^[^28^](#_ENREF_28)^)^ | 87.3 (80.5 –94)^(^[^6^](#_ENREF_6)^)^ | 5.8 (5.5–6)^(^[^6^](#_ENREF_6)^)^ | 40 | 12^(^[^28^](#_ENREF_28)^)^ |
| Pakistan | 91,000^(^[^29^](#_ENREF_29)^)^–423,000^(^[^30^](#_ENREF_30)^)^^[[10]](#footnote-10)^ | 27.2^(^[^29^](#_ENREF_29)^)^ | 85 (75-92.9)^(^[^6^](#_ENREF_6)^)^^[[11]](#footnote-11)^ | 6.8 (6–7.5)^(^[^6^](#_ENREF_6)^)^ | 34^(^[^31^](#_ENREF_31)^)^ | x |
| Philippines | 12,304–16,607^(^[^32^](#_ENREF_32)^)^ ^[[12]](#footnote-12)^ | 41.6^(^[^33^](#_ENREF_33)^)^ | 70^(^[^6^](#_ENREF_6)^)^ | nk | ✓^[[13]](#footnote-13)^ | x |
| Singapore | nk | nk | 42.5^(^[^6^](#_ENREF_6)^)^ | 8.5^(^[^6^](#_ENREF_6)^)^ | x | x |
| Sri Lanka | nk | nk | nk | nk | x | x |
| Taiwan | 60,000^(^[^34^](#_ENREF_34)^)^ ^[[14]](#footnote-14)^ | 17.7^(^[^34^](#_ENREF_34)^)^ | 41^(^[^6^](#_ENREF_6)^)^^[[15]](#footnote-15)^ | 16.7^(^[^6^](#_ENREF_6)^)^ | 1,103^(^[^22^](#_ENREF_22)^)^^[[16]](#footnote-16)^ | 90^(^[^22^](#_ENREF_22)^)^ (B, M) |
| Thailand | 40,300^(^[^35^](#_ENREF_35)^)^ | 25.2^(^[^36^](#_ENREF_36)^)^ | 89.8^(^[^6^](#_ENREF_6)^)^^[[17]](#footnote-17)^ | nk | 38^(^[^36^](#_ENREF_36)^)^ | 147 (M) |
| Vietnam | 271,000 (100,000–335,000)^(^[^37^](#_ENREF_37)^)^ | 10.3^(^[^37^](#_ENREF_37)^)^ | 74.1^(^[^6^](#_ENREF_6)^)^^[[18]](#footnote-18)^ | 19.5^(^[^6^](#_ENREF_6)^)^ | 297^(^[^37^](#_ENREF_37)^)^ | 80^(^[^37^](#_ENREF_37)^)^ |

1. UNAIDS. Global AIDS Response Progress Reporting: Islamic Republic of Afghanistan. Geneva: 2014.

2. (IBBS) IBBSS. Afghanistan Integrated Biological Behavioral Surveillance Survey (IBBS). Afghanistan: 2012.

3. Afghanistan MoPH. Program data. Afghanistan2014.

4. UNAIDS. Global AIDS Response Progress Reporting: Bangladesh. Geneva: 2014.

5. Crime UNOoDa. World Drug Report 2014. Vienna: 2014.

6. Nelson PK, Mathers BM, Cowie B, Hagan H, Des Jarlais D, Horyniak D, et al. Global epidemiology of hepatitis B and hepatitis C in people who inject drugs: results of systematic reviews. Lancet. 2011;378(9791):571-83.

7. KHANA N. Cambodia Integrated Biological Behavioral Surveillance Survey (IBBS). Cambodia: 2012.

8. Luo T. GSHR survey response 2014. AIDS Care China10 July 2014.

9. UNAIDS. Global AIDS Response Progress Reporting: China. Geneva: 2014.

10. Petersen Z, Pluddemann, A., van Hour, M., et al. The prevalence of HIV among people who inject drugs and availability of prevention and treatment services: findings from 21 countries. A brief report. Parow: 2012.

11. Organisation NAC. Ministry of Health & Family Welfare Annual Report 2012-13. India: 2014.

12. UNAIDS. Global AIDS Response Progress Reporting: India. Geneva: 2014.

13. Health NACaMo. Indonesia surveillance data. 2014.

14. UNAIDS. Global AIDS Response Progress Reporting: Indonesia. Geneva: 2012.

15. UNAIDS. Global AIDS Response Progress Reporting: Lao PDR. Geneva: 2014.

16. Bureau SW. Central Registration of Drug Abusers of Macau: Annual Report 2011. Macau: 2011.

17. Agrawal DA. Personal communication with Dr Alok Agrawal, Programme Officer, National AIDS Control Organisation (NACO), India. 1 May 2012.

18. Noguiera A. Personal communicaton with Augusto Noguira, President of the Association for Rehabilitation of Drug Abusers of Macau (ARTM). 18 May 2012.

19. UNAIDS. Global AIDS Response Progress Reporting: Malaysia. Geneva: 2012.

20. UNAIDS. Global AIDS Response Progress Reporting: Malaysia. Geneva: 2014.

21. UNAIDS. Global AIDS Response Progress Reporting: Maldives. Geneva: 2012.

22. Stoicescu C. The Global State of Harm Reduction 2012: Towards an integrated response. London: 2012.

23. Mathers BM, Degenhardt L, Phillips B, Wiessing L, Hickman M, Strathdee SA, et al. Global epidemiology of injecting drug use and HIV among people who inject drugs: a systematic review. The Lancet. 2008;372(9651):1733-45.

24. UNAIDS. Global AIDS Response Progress Reporting: Mongolia. Geneva: 2014.

25. UNAIDS. Global AIDS Response Progress Reporting: Myanmar. Geneva: 2012.

26. UNAIDS. Global AIDS Response Progress Reporting: Myanmar. Geneva: 2014.

27. Nepal MoHA. Central Bureau of Statistics. Nepal: 2013.

28. UNAIDS. Global AIDS Response Progress Reporting: Nepal. Geneva: 2014.

29. UNAIDS. Global AIDS Response Progress Reporting: Pakistan. Geneva: 2014.

30. UNODC. Drug Use in Pakistan 2013. Vienna: 2013.

31. Advisor SI. Personal communication with Strategic Information Advisor, UNAIDS Country Office for Pakistan and Afghanistan. August 2014.

32. Samonte G, Belimac, J., Feliciano, J. Philippine estimate of the most-at-risk population and people living with HIV. Philippine: 2011.

33. Health Do. Integrated HIV Behavioral & Serologic Surveillance. Philippines: 2013.

34. Huang Y, Yang, J., Nelson, K. et al. Changes in HIV Incidence among People Who Inject Drugs in Taiwan following Introduction of a Harm Reduction Program: A Study of Two Cohorts. PLoS Med. 2014;11(4).

35. al. Ae. Using the Multiplier Methods to Estimate the Population Size of Injecting Drug Users (IDUs) in Thailand. Republic of Korea: 2011.

36. UNAIDS. Global AIDS Response Progress Reporting: Thailand. Geneva: 2014.

37. UNAIDS. Global AIDS Response Progress Reporting: Vietnam. Geneva: 2014.

1. This includes all operational NSP sites, including fixed sites, vending machines and mobile NSPs operating from a vehicle or through outreach workers. [↑](#footnote-ref-1)
2. (M) = methadone, (B) = buprenorphine, (O) = any other form (including morphine and codeine). [↑](#footnote-ref-2)
3. Prevalence varies from 0.3% in Mazar city to 13.3% in Herat city. [↑](#footnote-ref-3)
4. Data from 2009, and only for men who inject drugs. [↑](#footnote-ref-4)
5. Based on data from Dhaka. [↑](#footnote-ref-5)
6. Figure indicates the number of registered people who use drugs that have been recorded by the police. There are an estimated 10 million people who use drugs thought to exist in China. [↑](#footnote-ref-6)
7. Data from 2008, with civil society organisations believing the actual figure to be much higher. [↑](#footnote-ref-7)
8. HCV prevalence varies greatly across the region, from 90% in Manipur to 1% in Bihar, but no national data is collected. [↑](#footnote-ref-8)
9. These are pilot NSPs in four districts of Houaphanh and Phongsaly province. [↑](#footnote-ref-9)
10. 91,000 figure based on a mapping methodology in 2009 in hotspots where people inject drugs. The 423,000 figure was derived from an epidemiological survey. Civil society recommended a range between both figures. [↑](#footnote-ref-10)
11. Figure from 2003. [↑](#footnote-ref-11)
12. Figure relates to adult males only. [↑](#footnote-ref-12)
13. Needles and syringes are distributed regularly to people who inject drugs but only at a health facility, thus limiting coverage. [↑](#footnote-ref-13)
14. Based on longitudinal data from two prison cohorts. [↑](#footnote-ref-14)
15. Figure from 2001. [↑](#footnote-ref-15)
16. Figure from 2005; no updated information on NSPs or OST in Taiwan. [↑](#footnote-ref-16)
17. Figure from 2000. [↑](#footnote-ref-17)
18. Figure from 2003. [↑](#footnote-ref-18)
